# Decreased MiR-30a promotes TGF-β1-mediated arachnoid fibrosis in post-hemorrhagic hydrocephalus

**DOI:** 10.1515/tnsci-2020-0010

**Published:** 2020-05-26

**Authors:** Chaohong Zhan, Gelei Xiao, Xiangyang Zhang, Xiaoyu Chen, Zhiping Zhang, Jingping Liu

**Affiliations:** Department of Neurosurgery, Xiangya Hospital, Central South University, Changsha, Hunan 410008, P. R. China; Diagnosis and Treatment Center for Hydrocephalus, Xiangya Hospital, Central South University, Changsha, Hunan 410008, P. R. China; National Clinical Research Center for Geriatric Disorders, Xiangya Hospital, Central South University, Changsha, Hunan 410008, P. R. China

**Keywords:** post-hemorrhagic hydrocephalus, fibrosis, miR-30a, TRAF3IP2, TGF-β1/smad3 signaling pathway

## Abstract

**Background:**

Fibrosis in the ventricular system is closely associated with post-hemorrhagic hydrocephalus (PHH). It is characterized by an expansion of the cerebral ventricles due to CSF accumulation following intraventricular hemorrhage (IVH). The activation of transforming growth factor-β1 (TGF-β1) may be involved in thrombin-induced arachnoid fibrosis.

**Methods:**

A rat model of PHH was established by injection of autologous non-anticoagulated blood from the right femoral artery into the lateral ventricles. Differential expression of miR-30a was detected in rat arachnoid cells by RNA sequencing. AP-1, c-Fos, and TRAF3IP2 were knocked down in primary arachnoid cells, and the degree of arachnoid fibrosis was assessed.

**Results:**

Decreased expression of miR-30a and increased expression of TRAF3IP2, TGF-β1, and α-SMA were detected in the arachnoid cells of PHH rat. Besides, overexpression of miR-30a targets TRAF3IP2 mRNA 3′UTR and inhibits the expression of TRAF3IP2, TGF-β1, and α-SMA in the primary arachnoid cells. Furthermore, TRAF3IP2 activates AP-1 to promote arachnoid fibrosis. The content of type I collagen in the primary arachnoid cells was reduced after the silencing of AP-1 and TRAF3IP2.

**Conclusions:**

This study identified a miR-30a-regulated mechanism of arachnoid fibrosis, suggesting a previously unrecognized contribution of miR-30a to the pathogenesis of fibrosis in the ventricular system. These results might provide a new target for the clinical diagnosis and treatment of PHH.

## 1 Introduction

Post-hemorrhagic hydrocephalus (PHH) is a serious complication of intraventricular hemorrhage (IVH), subarachnoid hemorrhage (SAH), and traumatic brain injury (TBI). It has a high mortality and morbidity [1,2,3,4,5]. Failure to treat PHH immediately may lead to various complications, such as loss of consciousness, dementia, and persistent coma, seriously threatening the safety of patients [6]. At present, cerebrospinal fluid shunt surgery is the main treatment for PHH. However, patients need multiple surgeries, which require high levels of manpower and material resources because of complications such as infection or shunt tube blockage and fracture. Therefore, it is crucial to identify novel therapeutic targets and to develop new targeted drugs for hydrocephalus. Of note, the pathogenesis of PHH should be intensively investigated to promote the treatment of PHH etiologically [7,8].

Studies have shown that the occurrence and development of PHH are closely related to fibrosis, which is always associated with the obstruction of CSF circulation [9,10]. Fibrosis refers to the pathological process of parenchyma cell necrosis and the abnormal increase and excessive deposition of extracellular matrix (ECM) due to inflammation and other factors. Zhang et al. also observed the excessive deposition of ECM in PHH [11], which suggested that fibrosis in arachnoids played a critical role in the occurrence and development of PHH. Then, it has a potential clinical value to study the role of arachnoid fibrosis after intracerebral hemorrhage.

PHH is mainly induced by IVH. Vascular endothelial damage and tissue factor release in the process of ventricular hemorrhage can activate various coagulation factors and induce blood hypercoagulation or thrombus formation. Thrombin is one of the important coagulation factors in this process. Studies have shown that coagulation enzymes play an important role in the occurrence and development of PHH. A PHH model can be successfully constructed by direct injection of thrombin into the ventricle of a rat [12]. In addition to coagulation function, thrombin also plays an important role in the pathological process of cellular fibrosis [13]. In hepatic stellate cells, thrombin binds to protein-activated receptor 1 (PAR-1) and activates downstream signaling pathways to cause fibrosis [14]. Besides, thrombin can promote the formation of fibrin clots in the liver and catalyze the conversion of soluble fibrin into fibrin monomers to aggravate liver fibrosis [15].

The activation of transforming growth factor-β1 (TGF-β1) induced by thrombin during PHH is the main cause of arachnoid fibrosis. TGF-β1 was originally isolated from platelets and activated under abnormal conditions. In some pathological conditions, TGF-β1 was activated and released into CSF to cause fibrosis [16,17]. Injection of TGF-β1 into the brain could induce the deposition of many collagen fibers in the cell space and arachnoid convex area of the meningeal membrane [18]. Meanwhile, the expression of TGF-β1 in hemorrhagic hydrocephalus was higher than that in non-hemorrhagic hydrocephalus [19]. Thrombin could induce TGF-β1 activation after activating its receptor PAR-1. Therefore, the TGF-β1 signaling pathway may be a potential target for inhibiting arachnoid fibrosis.

miRNAs, a type of noncoding RNA between 19 and 25 nucleotide (nt), can act as a kind of post-transcriptional inhibitor. Approximately 30% of human genes are negatively regulated by miRNAs at transcriptional or post-transcriptional levels. These molecules mediate post-translational inhibition by binding complementary sequences of target gene transcripts or promoting the dissociation of target mRNA, which eventually leads to target gene silencing. Increasing evidence suggests that miRNAs were involved in fibrosis. miR-133a has been proven to protect the myocardium from fibrosis [20]. TGF-β1 promotes collagen expression and renal fibrosis by inhibiting miR-29 [21]. Liver fibrosis led to the downregulation of miR-150 and miR-194, resulting in the inactivation of hepatic stellate cells [22].

In the present study, we hypothesized that miR-30a may be the potential therapeutic targets in PHH. Overexpression of miR-30a may be able to inhibit arachnoid fibrosis. By using miRNA analysis and real-time PCR, we identified that loss of miR-30a was associated with arachnoid fibrosis in an experimental rat model of PPH. We also identified that TRAF3IP2 was a target of miR-30a. In a rat model of PPH, arachnoid fibrosis was associated with the upregulation of TRAF3IP2 when the mRNA and protein expression levels of miR-30a were decreased. In contrast, overexpression of miR-30a was capable of suppressing TRAF3IP2, thereby inhibiting TGF-β1-mediated arachnoid fibrosis *in vitro* and *in vivo*.

## 2 Methods

### 2.1 Animals

A total of 30 adult male Sprague-Dawley rats weighing 250–350 g were provided by the Hunan SJA Laboratory Animal Co., Ltd. Animals were randomly divided into two groups: the PHH model group and the control group. These animals were housed under controlled and pathogen-free conditions in which they had free access to water and pellet food.


**Ethical approval:** The research related to animal use has been complied with all the relevant national regulations and was approved by No. 20180699 of the Xiangya Animal Protection and Utilization Committee of Central South University for the care and use of animals.

### 2.2 PHH model protocols

The PHH model was constructed based on previous publications (Supplementary Figure 1) [23]. Briefly, animals were anesthetized with 10% chloral hydrate (400 mg/kg intraperitoneal) and fixed in a stereotaxic frame. The body temperature of the rats was maintained at 37.0°C with a feedback-controlled heating pad. A cranial hole (1 mm) was drilled (coordinates: 1.6 mm lateral and 0.8 mm posterior to the bregma), and a micromanipulator was inserted at a depth of 3.2 mm from the dura. One hundred microliters of autologous arterial blood drawn from the right femoral artery was microinjected at a rate of 10 μL/min using a microinfusing pump in each rat. The sham groups only underwent needle injection. After the procedures, the hole was covered with bone wax, and the skin incisions were closed by sutures.

### 2.3 HE staining

Paraffin sections of rat brain tissue were dewaxed and hydrated using xylene and ethanol. The samples were stained with hematoxylin for 3 min. Then, the floating color was washed off by hydrochloric acid/ethanol solution to differentiate for 1.3 s. The samples were rinsed with running water for 15 min. After that, slices were dyed with eosin for 1–3 min and washed away from the floating color.

### 2.4 Masson staining

Brain tissue with meninges was cut in a coronal position with a thickness of 10 µm, fixed in Bouin’s solution for 2 h, rinsed with running water until no yellow was observed, and then dried at room temperature. Masson staining was performed in strict accordance with the instructions. The field of vision at the top and the lateral margin at a time of 400 was selected, and the soft meninges gray value and thickness were measured by Image-Pro Plus software; (100× thickness)/gray value was used as the fibrosis index.

### 2.5 Magnetic resonance imaging and ventricle volume measurement

Rats were anesthetized with 10% chloral hydrate for MRI examination. MRI was performed by a 7.0-T Varian MR scanner (GE, USA) with a T2 sequence using a view field of 35 mm × 35 mm and 25 coronal slices. Bilateral ventricles were outlined on a computer, and the areas were measured. Image analysis was performed using the MRiLab1.0a program by a masked observer.

### 2.6 Cell isolation and culture

To isolate arachnoid cells, the rats were euthanized with 10% chloral hydrate (400 mg/kg intraperitoneal). Pia-arachnoid tissue was harvested from the anterior portion of the brainstem by microsurgical techniques and washed three times with ice-cold PBS. Then, the tissue was microdissected from extraneous tissue and chopped into 1 × 1 mm^2^ pieces. These tissue fragments were incubated in a humidified atmosphere of 5% CO_2_ at 37°C in culture media containing Eagle’s DMEM and 10% FBS (Shanghai Weijin Biotechnology Co., Ltd), nonessential amino acids (Solarbio, Beijing, China), glutamine (Solarbio, Beijing, China), and streptomycin–penicillin (Solarbio, Beijing, China) at 10 µL/mL. The media were changed for three times per week. Within 3–4 days, cells grow out of the dissected tissue and were confluent at 10–15 days. Cells were passaged following trypsinization with PBS mixed with a 1:2 dilution of 0.02% EDTA–0.05% trypsin (Solarbio, Beijing, China).

### 2.7 ELISA

The CSF of the PHH rat and control rat was separately collected for the detection of TGF-β1 using an ELISA kit. For TGF-β1 detection, 1 mol/LHCl was added to the CSF at a ratio of 3:1 and incubated for 10 min to activate TGF-β1. After neutralization with 1 mol/L NaOH/0.5 MHEPES, the sample was then analyzed with an ELISA kit to detect the level of TGF-β1. Besides, after stimulation with the various treatments, arachnoid cells culture conditioned media (supernatants) were collected. ELISA kit was used to detect the TGF-β1 level (EK0514, Wuhan Boster Biological Technology, Ltd, China), collagen type I level (CSB-E08084r, CUSABIO, China), and collagen type III level (CSB-E07924r, CUSABIO, China) following the protocols constructions.

### 2.8 Lentiviral vector construction and transfection

The AP1 shRNA, control shRNA, and corresponding lentiviruses were purchased from GeneChem Ltd. (Shanghai, China). The AP1 shRNA target sequence was 5′-TGGTGGCGTTCGTTTC-3′, the TRAF3IP2 shRNA target sequence was as follows: 5′-CCGGGCTTCAGAACACTCATGTTTACTCGAGTAAACATGAGTGTTCTGAAGCTTTTTG-3′. The sequences of control shRNA were as follows: 5′-UUCUCCGAACGUGUCACGU-3′. For transfection, arachnoid cells were seeded into six-well plates and then infected with the constructed AP1 shRNA lentiviruses and control shRNA lentiviruses at an MOI of 30. After 8 h, the supernatant was moved, and fresh medium was added. Then, the cells were put back into the incubator for 96 h. The lentiviral vectors can express GFP, which can be used for the measurement of infection efficiency in arachnoid cells.

### 2.9 Transfection of miRNA mimics, inhibitor

Synthetic miR-30a or inhibitor was transfected into arachnoid cells using INTERFER (Polyplus transfection) according to the standard protocol. The sequences of miR-30a were as follows: sense, 5′-UGUAAACAUCCUCGACUGGAAG-3′, antisense, 5′-CUUCCAGUCGAGGAUGUUUACA-3′; the sequence of the 2′-*O*-methyl-modified miR-30a inhibitor was as follows: 5′-CUUCCAGUC GAGGAUGUUUACA-3′.

### 2.10 Fluorescence

Fluorescence pictures were acquired with a 200 M microscope (Zeiss Axiovert, Germany) with FITC/Alexa Fluor 488, filters for DAPI, DsRed/PE, Texas Red/Alexa Fluor 568/594, and Zeiss Axio Vision software. Exposure times were adjusted separately and kept for the complete session for each channel. Adjustment of brightness and contrast was performed separately.

### 2.11 RNA preparation, reverse transcription, and quantitative real-time PCR

Total RNA was prepared from cultured cells using TriZol reagent (Invitrogen) according to the manufacturer’s instruction [24]. Total RNA was reverse transcribed with random primers and oligo dT (18 mers) (Agilent Technologies). The quantitative real-time PCR reactions were performed by SYBR Green qPCR Master Mix (MedChemExpress, China) on a Rotor-GeneTM 3000 real-time PCR machine (Microspeed Biotechnology, Shanghai, China). The specific primers forTRAF3IP2 mRNA and TGF-β1 mRNA were as follows: TRAF3IP2, 5′-TGGCACCCAACAGCTTGTC-3′ (forward) and 5′-GATACAGGCCGCTGGTGATTT-3′ (reverse); TGF-β1, 5′-GGCGATACCTCAGCA ACCG-3 (forward) and 5′-CTA AGG CGA AAG CCC TCA AT-3′ (reverse). The initial denaturation was performed for 5 min at 95°C following 50 cycles of denaturation for 10 s at 95°C. Eventually, the extension was performed at 73°C for 20 s under primer-specific conditions. Comparative quantitation was performed in selected groups using the Relative Expression Software Tool (Relative Expression Software Tool, Qiagen), which is based on the Pair Wise Fixed Reallocation Randomization Test©.

### 2.12 RNA analysis

Small RNA deep sequencing was performed and analyzed as previously described [25]. RNA was extracted from the arachnoid cells of control rats and PHH model rats as previously described. Total RNA was isolated, quality-checked, and quantified. Two sRNA libraries were constructed. Following the manufacturer’s instructions (Illumina), the small RNAs, with a size of 18–31 nt, were excised from total RNA by polyacrylamide gel electrophoresis (PAGE) purification. After that, the sRNAs ligated to a pair of adaptors at the 5′ and 3′ ends. The sRNAs with the adapters in place were amplified by RT-PCR. Last, the generated cDNA libraries were sequenced by Illumina HiSeq 2000 sequencing technology (Berry Genomics Biotech, Beijing, China). Bioinformatics methods were used to identify the miRNAs. First, small sequences (sizes  < 18 nt), adapter sequences, contaminated reads, and low-quality reads were removed. Clean reads with a length of 18–31 nt were accounted. Second, clean reads were compared with the Rfam database (http://rfam.sanger.ac.uk/) to match with the known rRNA, small nuclear RNA (snRNA), small nucleolus RNA (snoRNA), and tRNA sequences. After these non-coding RNAs were deleted, the remaining sRNAs were matched with the pre-miRNAs database in miRbase. These reads were used to identify mature miRNAs. Finally, differential expression of arachnoid miRNA between control rats and PHH rats was assessed by applying false discovery rate-corrected thresholds (*P*-value < 0.05).

### 2.13 miRNA RT-qPCR

Total miRNAs were prepared from cultured cells using TRIzol reagent (Thermo) and phenol–chloroform extraction. Specific miRNA primers formiR-30a was as follows: 5′-ACACTCCAGCTGGGTGTAAACATCCTCGAC-3′ (forward) and 5′-CAGTGCGTGTCGTGGAGT-3′ (reverse). The miRcute miRNA cDNA kit (Tiangen Biotech Co., Ltd) was used to reverse transcribe cDNA, and the miRcute Plus miRNA qPCR Kit (Tiangen Biotech Co., Ltd) was used to detect RT products. The thermal cycling conditions were as follows: 95°C for 5 min, followed by 40 cycles at 95°C for 15 s, 60°C for 30 s, and 72°C for 20 s. The relative expression was calculated with the 2^−ΔΔCt^ method.

### 2.14 Western blot analysis

Proteins were separated by SDS-PAGE and then transferred to PVDF membranes (Haoran Biotechnology, Shanghai, China) by a Trans-Blot SD Semi-Dry Electrophoretic Transfer Cell (Bio-Rad) according to previous publications [26]. Membranes were blocked in 1× PBS containing 0.1% Tween 20 and 5% skim milk for 1 h at 25°C and then incubated with primary antibody by 1× PBS containing 0.1% Tween 20 and 2% skim milk overnight at 4°C. Next, the membranes were washed three times for 5 min with 0.1% Tween 20 solution in 1× PBS followed by incubation with secondary antibody for 1 h at 25°C in 1× PBS containing 0.1% Tween 20 and 2% skim milk. The membranes were washed three times for 5 min. Chemiluminescence signals were detected by Super Signal West FemtoChemiluminescence Substrate (Thermo Scientific) and a LAS 4000 Bioimager (GE Healthcare). The following antibodies were used: α-SMA (17H19L35, eBioscience, USA), TRAF3IP2 (IMG-563, San Diego, CA), GAPDH (sc-365062, Santa Cruz, USA), TGF-β1 (BS-1361, Bioworld Biotechnology Co, USA), c-Fos (ab209794, Abcam, USA), and AP-1 (PL0302152, PLLABS, CA).

### 2.15 Statistical analysis

The groups were compared by one-way analysis of variance (ANOVA) followed by a specific *post hoc* test. Statistical analysis was performed using Relative Expression Software Tool 2009 (Relative Expression Software Tool, Qiagen, Hilden, Germany), which is based on the Pair Wise Fixed Reallocation Randomization Test© (Pfaffl et al., 2002). Differences were considered statistically significant for *p < 0.05, **p < 0.01, and ***p < 0.001.

## 3 Results

### 3.1 Elevated expression of TGF-β1 in ratPHH model

**Figure 1:**
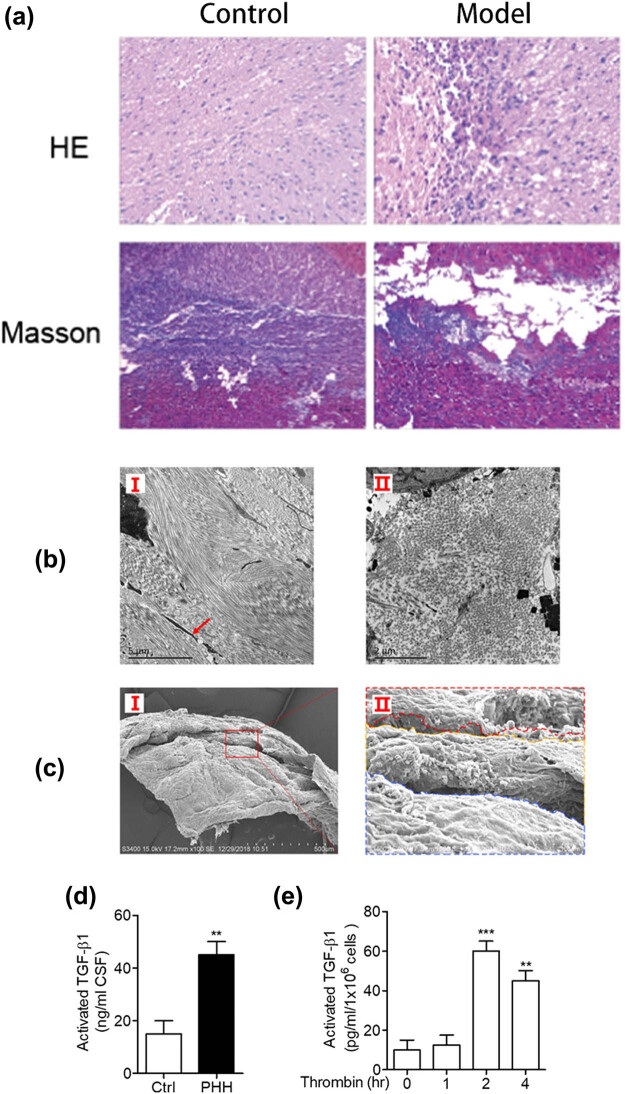
Identification of PHH rat model. (a) Results of HE and Masson staining in the brain tissues showed that the cerebral cortex was compressed and thinned, and the cells of subcutaneous brain tissue were swollen and deformed. (b) Transmission electron microscopy of arachnoid in PHH rats: Figure I shows the longitudinal collagen fibers in the arachnoid, presenting dense collagen fibers and complete arachnoid fibrosis. The red arrow shows fibroblasts, suggesting that fibrosis may occur in arachnoid cells. Figure II shows the horizontal collagen fibers in the arachnoid of the rats, presenting dense collagen fibers and arachnoid fibrosis. (c) Tomography of brain tissues in PHH rats. Figure II is a partial magnification schematic diagram of the red box in Figure I, presenting obvious fibrosis in the arachnoid and subarachnoid space. The subarachnoid space was filled with collagen fibers, and the pores of the arachnoid cerebellum were narrowed. (d) Detection of activated TGF-β1 in the CSF by ELISA (**P < 0.01 compared with the control group; *P < 0.05 compared with the control group). (e) Thrombin can promote the activation of TGF-β1 in primary arachnoid cells. The concentration of activated TGF-β1 was detected by ELISA after treated with thrombin (10 U/mL) for different times (**P < 0.01 compared with the control group; ***P < 0.001 compared with the control group).

A rat model of PHH was constructed and confirmed though HE staining, Masson staining, and transmission electron microscopy. In the PHH group, the cerebral cortex of rats was compressed and thinned (Figure 1a), and high-density collagen fibers could be observed in arachnoid (Figure 1b). The subarachnoid space was filled with collagen fibers, and the pores of the arachnoid cerebellum were narrowed (Figure 1c). We found that the expression of TGF-β1 is elevated in the PHH model. The content of activated TGF-β1 in the cerebrospinal fluid was significantly upregulated (Figure 1d). The activated TGF-β1 level in the primary arachnoid cells of PHH rats was also higher than that in the control group after treatment with thrombin (Figure 1e).

### 3.2 TRAF3IP2 activates AP-1 to promote arachnoid fibrosis

**Figure 2:**
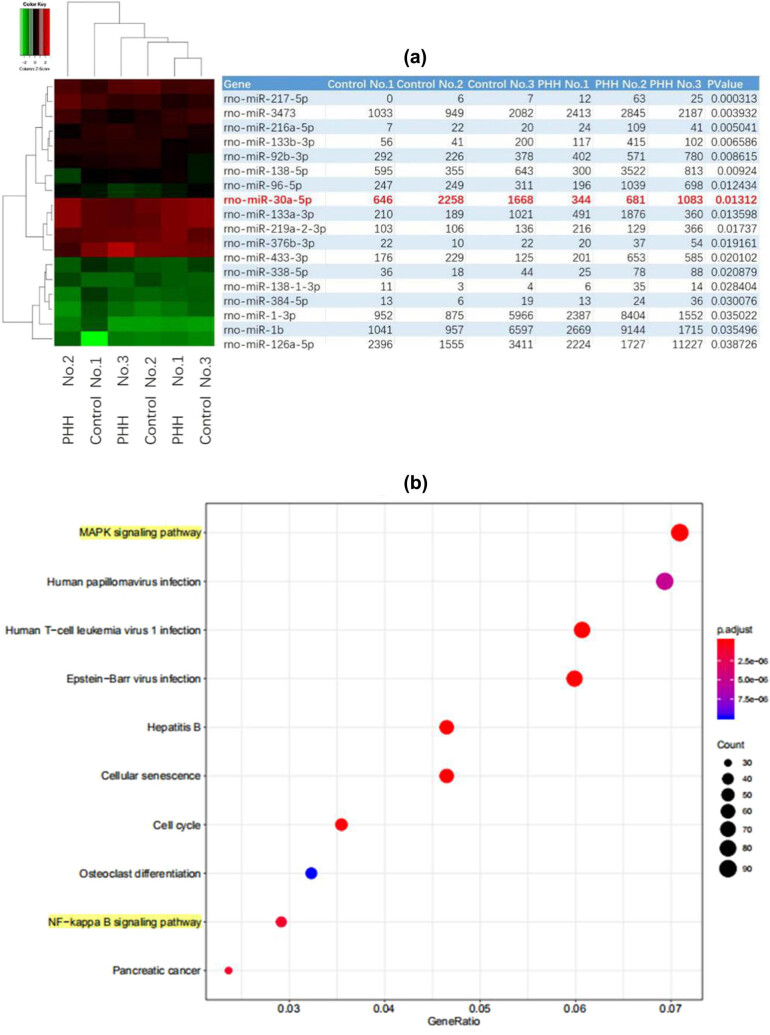
MiR-30a can target TRAF3IP2 and participate in the regulation of arachnoid fibrosis. (a) Differential expression of arachnoid miRNAs between control rats and PHH rats. (b) KEGG miRNA enrichment diagram shows that the NF-κB signaling pathway was activated, suggesting that TRAF3IP2 may be activated.

We determined the differential expression miRNAs in the arachnoid cells of the PHH model. Eighteen miRNAs, including miR-30a, were differentially expressed, and the expression of miR-30a was significantly decreased (Figure 2a). Moreover, there was obvious activation of NF-κB signaling pathway in PHH rats (Figure 2b), and TRAF3IP2 expression may be induced by NF-κB because its promoter region contains a potential binding site for NF-κB, which indicated that the TRAF3IP2-related pathway was activated after TGF-β1 activation induced by thrombin.

**Figure 3:**
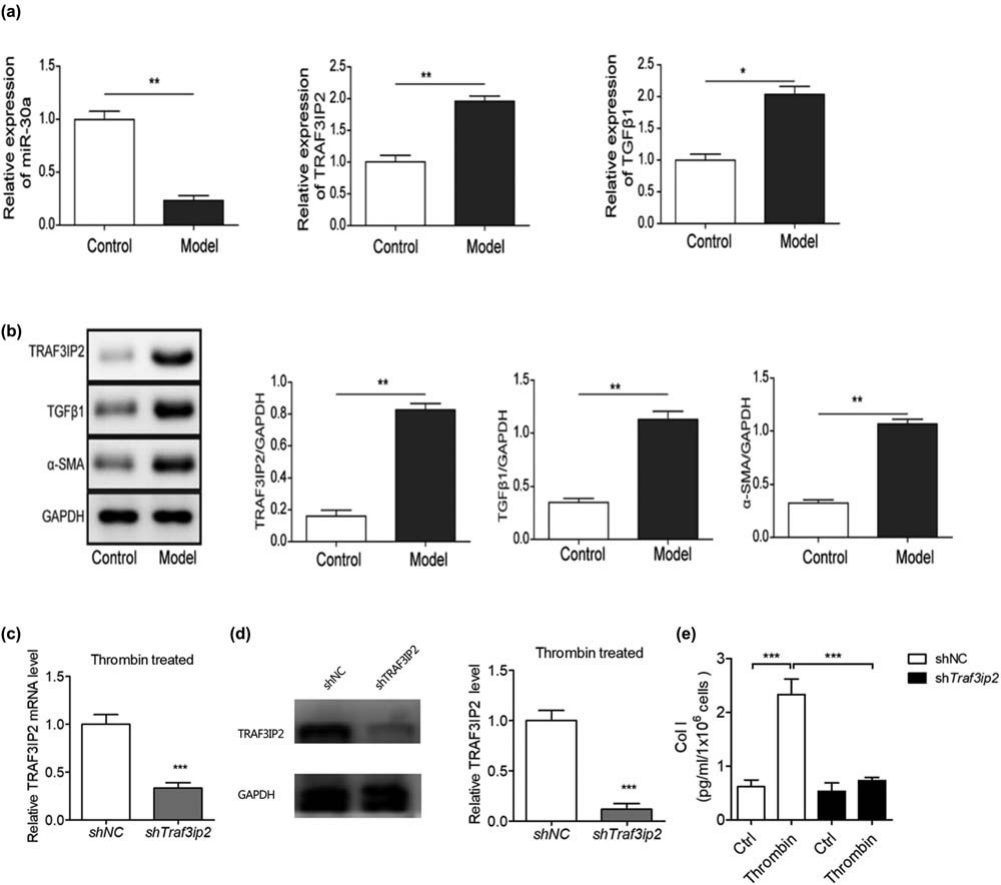
TRAF3IP2 activates AP-1 which promotes arachnoid fibrosis. (a) Differential mRNA expression of miR-30a, TRAF3IP2, and TGF-β1 between PHH rats and control rats (**P < 0.01 compared with the control group; *P < 0.05 compared with the control group). (b) Differential protein expression of TRAF3IP2, TGF-β1, and α-SMA between PHH rats and control rats (*P < 0.05 compared with the control group). (c) Decreased expression of TRAF3IP2 mRNA after transfected with TRAF3IP2 shRNA lentivirus (***P < 0.001compared with the control group). (d) Silenced expression of TRAF3IP2 protein after transfected with TRAF3IP2 shRNA lentivirus (***P < 0.001 compared with the control group). (e) Content of Collagen I in the primary arachnoid cells, which were transfected with TRAF3IP2 shRNA lentivirus for 48 h and treated with 10 U/mL thrombin for 4 h (***P < 0.001 compared with the control group).

We also measured the differential expression of miR-30a, TRAF3IP2, and related genes in arachnoid cells between the PHH group and control group. The expression of miR-30a was decreased and the expression of TRAF3IP2, TGF-β1, and α-SMA was upregulated in the PHH group, suggesting that miR-30a and TRAF3IP2 may be involved in arachnoid fibrosis (Figure 3a and b).

To further study whether TRAF3IP2 induce arachnoid fibrosis during PHH, we knocked down TRAF3IP2 in primary arachnoid cells and determined the degree of arachnoid fibrosis. The results showed that collagen I, a marker of fibrosis, decreased significantly after knockdown of TRAF3IP2, indicating that the knockdown of TRAF3IP2 can reduce the level of subarachnoid cell fibrosis due to the activation of TGF-β1 (Figure 3c–e). This finding indicates that thrombin-induced TGF-β1 activation in PHH may promote subarachnoid fibrosis by upregulating TRAF3IP2.

### 3.3 Blocking AP-1 reduces arachnoid fibrosis mediated by TGF-β1 during PHH

**Figure 4:**
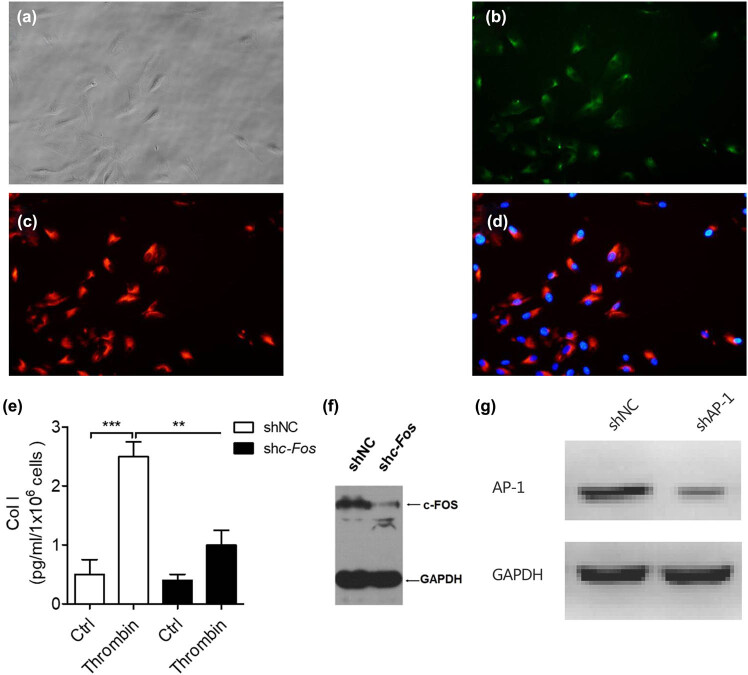
Blocking AP-1 can reduce the fibrosis of primary arachnoid cells induced by thrombin. (a) Rat primary arachnoid cells. (b) Desmoplakin staining and green fluorescent labeling. (c) Cytokeratin staining and red fluorescent labeling. (d) Double fluorescence merging. (e) Expression of c-FOS in primary arachnoid cells was detected by immunoblotting after knocking down c-FOS (***P < 0.001 compared with the control group). (f) Expression of AP-1 in primary arachnoid cells was detected by immunoblotting after transfected with AP-1 shRNA lentivirus (***P < 0.001 compared with the control group). (g) Changes in type I collagen content in the primary arachnoid cells, which were transfected with c-FosshRNA with lentivirus for 48 h and treated with 10 U/mL thrombin for 4 h (**P < 0.01 compared with the control group; ***P < 0.001 compared with the control group).

To study the possible molecular mechanism of arachnoid fibrosis during PHH, the primary arachnoid cells of rats were isolated, cultured, and identified (Figure 4a–d). TRAF3 interaction protein 2 (TRAF3IP2) is the upstream regulatory factor of activator protein-1 (AP-1). AP-1 is a transcription activation factor which can combine with the Smads complex containing c-Fos and c-Jun. To block the function of AP-1 in the primary arachnoid cells of rats, we transfected it with c-Fos shRNA lentivirus (Figure 4e) and shAP-1 shRNA lentivirus (Figure 4f) separately and detected its expression by immunoblotting. After 48 h, we treated it with 10 U/mL thrombin for 4 h and observed the downregulation of collagen I expression in arachnoid cells (Figure 4g), indicating that the antifibrotic effect of arachnoid cells was enhanced.

### 3.4 MiR-30a blocks arachnoid cell fibrosis by inhibiting TRAF3IP2 *in vivo*

**Figure 5:**
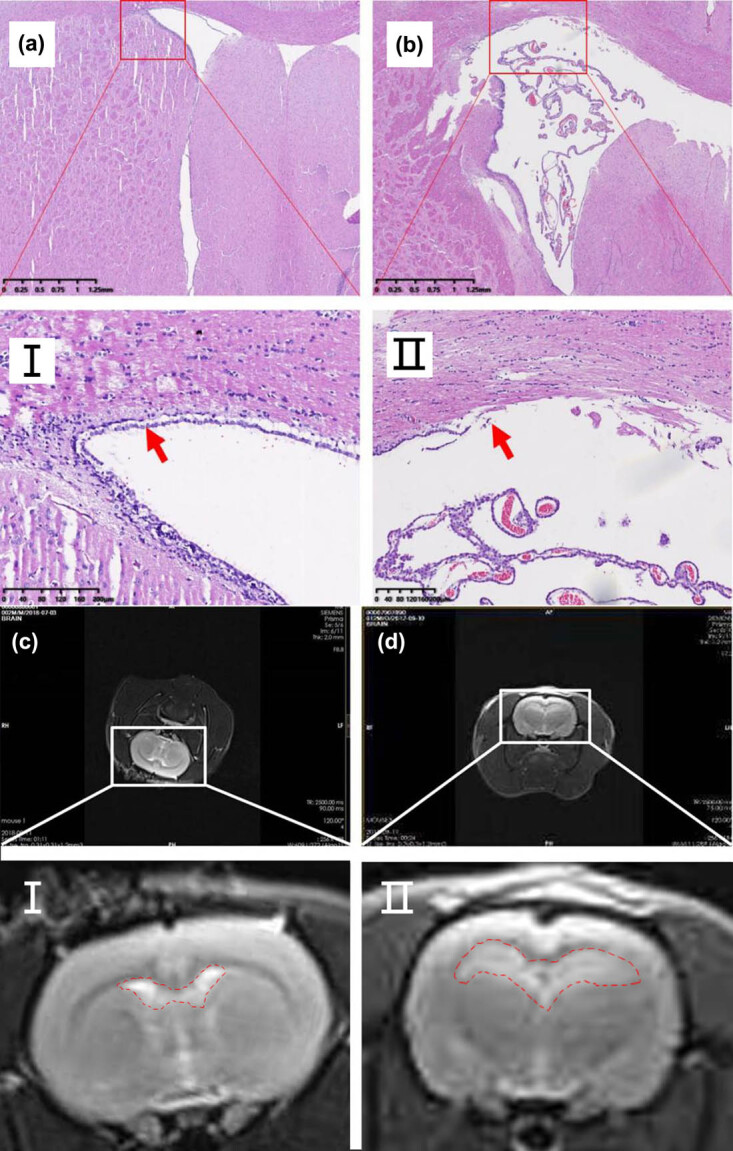
MiR-30a inhibits the formation of hydrocephalus induced by IVH in rats. (a) In the miR-30a overexpressing group, the morphology of the ventricle is close to the normal structure. The ventricular wall structure was complete, and the ventricular membrane cells and the ciliary morphology were normal (red arrow in (I)). (b) In the control group, the ventricle of rats was significantly enlarged, and there was hemorrhage in the choroid plexus of the ventricle. Partial magnification (II) from b shows that the structure of the ventricular wall was destroyed, ependymal cells were distorted and deformed, and some cilia were lost (II red arrow). (c and d) A comparison of MRI results in two groups of rats. The morphology of the ventricle in the miR-30a overexpression group is close to normal. However, in the control group, the ventricle of PHH rats was significantly enlarged, the cerebral cortex became thinner, the ventricle was enlarged, the angle of ventricle became obtuse, and there was obvious hydrocephalus.

To clarify the effect of miR-30a on PHH formation, two groups of rats were subjected to IVH according to the above method. HE results showed that the ventricle of the control group was significantly larger than that in the miR-30a overexpressing group (Figure 5a and b). MRI results showed that the ventricle was enlarged, the cerebral cortex became thinner in the saline group, but the morphology of the ventricle is close to normal in the miR-30a overexpressing group (Figure 5c and d). The formation rate of PHH in the miR-30a overexpression group was lower than that in the control group, and the changes in ventricle morphology and ventricle structure were better than those in the control group. These results suggest that miR-30a may inhibit and slow the formation of hydrocephalus.

### 3.5 Decreased MiR-30a induced arachnoid cell fibrosis through TRAF3IP2 *in vitro*

To verify whether miR-30a regulates the TGF-β1/smad3 signaling pathway through TRAF3IP2 in subarachnoid fibrosis and PHH formation, we used thrombin-induced arachnoid fibrosis *in vitro* model and compared the expression of miR-30a and related fibrosis indexes between the two groups.

**Figure 6:**
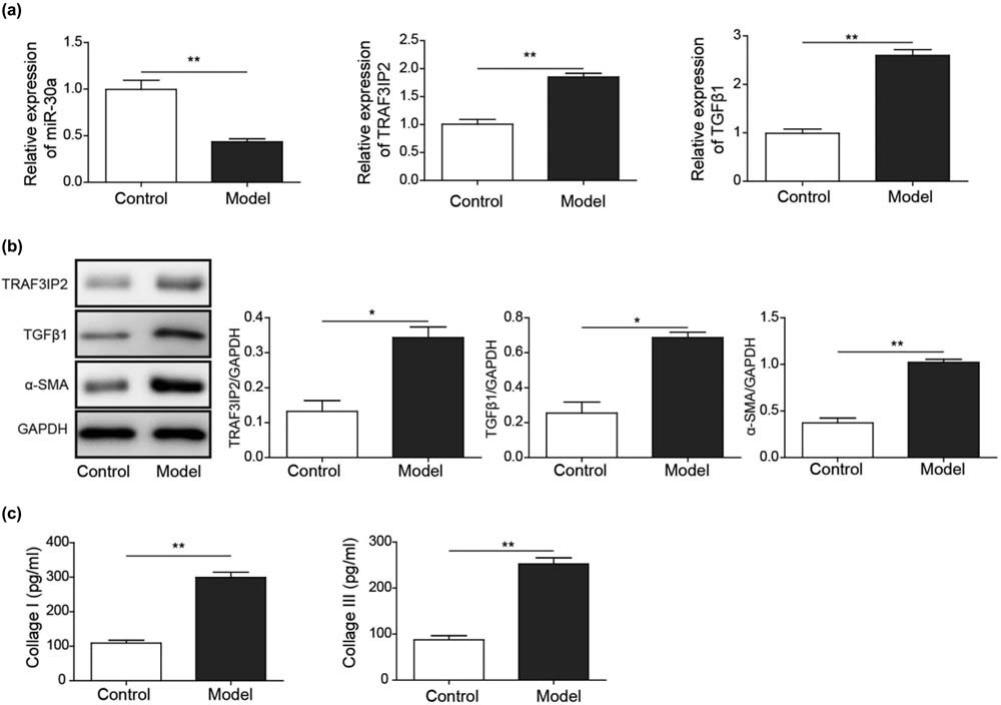
In the *in vitro* model of arachnoid cell fibrosis, the expression of miR-30a decreased, and the expression of TRAF3IP2 was upregulated. (a) Differential mRNA expression of miR-30a, TRAF3IP2, and TGF-β1 in arachnoid cells between the fibrotic group and control group (**P < 0.01 compared with the control group). (b) Differential protein expression of TRAF3IP2, TGF-β1, and α-SMA in arachnoid cells between the fibrotic and control groups (**P < 0.01 compared with the control group; *P < 0.05 compared with the control group). (c) Differential content of collagen I and collagen III in the model of arachnoid cell fibrosis (**P < 0.01 compared with the control group).

In the thrombin model group, the expression of miR-30a decreased, while the expression of TRAF3IP2 and TGF-β1 was upregulated (Figure 6a). The protein expression levels of TRAF3IP2, TGF-β1, and α-SMA were also upregulated at the same time (Figure 6b). Besides, we also detected the upregulated content of collagen I and collagen III in the thrombin model group (Figure 6c), suggesting that decreased expression of miR-30a may be engaged in the fibrosis of arachnoid cells in subarachnoid fibrosis and PHH formation.

**Figure 7:**
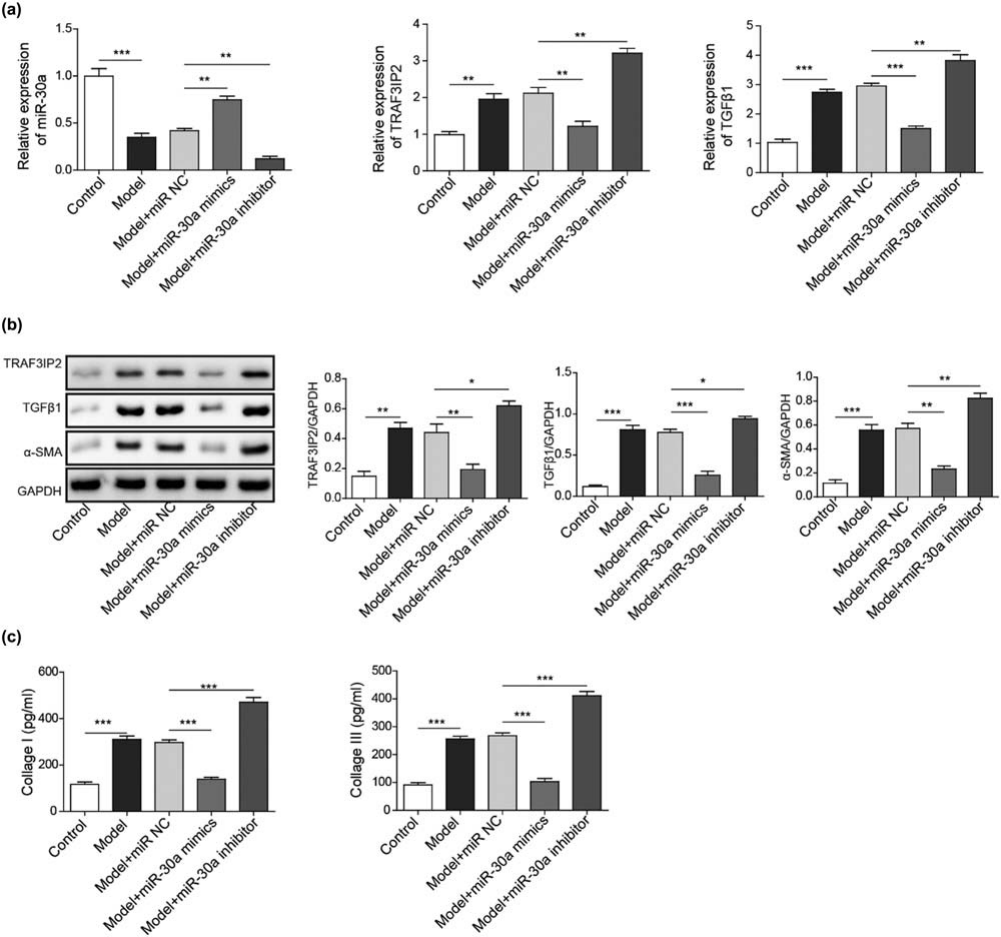
MiR-30a inhibits subarachnoid fibrosis through inhibiting TRAF3IP2. (a and b) The mRNA and protein expression of miR-30a, TRAF3IP2, TGF-β1 and α-SMA from different groups (**P < 0.01 compared with the control group; ***P < 0.001 compared with the control group). (c) The content of collagen I and collagen III from different groups (***P < 0.001 compared with the control group. **P < 0.01 compared with the control group; *P < 0.05 compared with the control group).

Overexpression of miR-30a and inhibition of miR-30a were constructed for the further experiment (Figure 7a). The mRNA level of TRAF3IP2 and TGF-β1 was decreased following the transfection of miR-30a mimics, and the mRNA level of TRAF3IP2 and TGF-β1 was increased following the transfection of miR-30a inhibitor (Figure 7b). In parallel, the protein level of TRAF3IP2, TGF-β1, and α-SMA was inhibited in the miR-30a mimics group and upregulated in the miR-30a inhibitor group (Figure 7b). In addition, the content of collagen I and collagen III was also decreased in the miR-30a mimics group and increased in the miR-30a inhibitor group (Figure 7c).

## 4 Discussion

TGF-β1 plays an important role in the formation of hydrocephalus [27]. Activated TGF-β1 can initiate a series of intracellular signal cascade reactions, including the *p38* MAPK pathway, ERK/MAPK pathway, and Smad pathway [28,29,30], which further increases the synthesis of collagens, other ECM components, and arachnoid fibrosis. Activation of thrombin increased TGF-β1 in the cerebrospinal fluid [12,31]. The TRAF3IP2/AP-1 axis is important in the process of tissue cell fibrosis and TRAF3IP2 mediates the activation of AP-1 [32]. For the first time, this study demonstrated the involvement of miR-30a in hydrocephalus development and confirmed that miR-30a affects TRAF3IP2/AP-1 signaling in thrombin-induced hydrocephalus formation. Thrombin induces TGF-β1 expression and activates the TRAF3IP2/AP-1 signaling pathway which ultimately promotes arachnoid fibrosis.

TGF-β1/smad pathways and other TGF-β1-related pathways were associated with fibrosis. TGF-β1 was increased in the cerebrospinal fluid of rats with hydrocephalus, and TGF-β1 expression in the arachnoid cell was also higher than that in the control group. These findings suggested that the involvement of TGF-β1 in the development of PHH and arachnoid fibrosis. Consistent with these previous studies of TGF-β1, our results not only found that TRAF3IP2 changes parallel to TGF-β1 in both cerebrospinal fluid and arachnoid cells, but also that TRAF3IP2 can activate the NF-κB and AP-1 pathways [33].

It has been shown that blocking AP-1 transcription inhibits the activation of fibrosis and reduces the production of fibrotic mediators [34]. Our results also showed that TGF-β1-mediated arachnoid fibrosis in PHH was attenuated by shRNA inhibition of the transcription factor AP-1. This finding indicates that AP-1 engaged in the occurrence of PHH mediated through TGF-β1, which is regulated by TRAF3IP2. Furthermore, TGF-β1 and TRAF3IP2 also increased in the process of arachnoid cell fibrosis. Therefore, after SAH, the activation of thrombin led to an incrassation in TGF-β1, which further induce the activation of the TRAF3IP2/AP-1 axis and finally lead to arachnoid fibrosis and hydrocephalus. However, we also found that the NF-κB signaling pathway was also activated at the same time when TRAF3IP2 was activated. Therefore, in addition to the AP-1 pathway, TRAF3IP2 may also induce arachnoid fibrosis through the NF-κB signaling pathway, which still needs more work to verify [35].

Noncoding RNAs are essential regulators of cellular signaling which are closely related to hydrocephalus and fibrosis. A previous study [36] reported the expression profiles of lncRNAs in an animal model with hydrocephalus, and a total of 1,575 lncRNAs were differentially expressed in hydrocephalus. Many of these lncRNAs are associated with inflammation and immune responses, such as NR5A2, H19, and NONMMUT127892.1. This finding suggests that the occurrence and development of hydrocephalus may be related to the differential expression of noncoding RNAs. At the same time, lncRNAs have been identified to have both promotive and inhibitory effects in the processes of fibrosis [37]. Liu et al. [38] discovered that cholangiocyte-derived exosomallncRNAH19 can promote cholestatic liver fibrosis. Other studies also showed that circulating miR-103a-3p leads to angiotensin II-induced renal inflammation and fibrosis through an SNRK/NF-κB/p65 regulatory axis. LncRNAAK081284, miR-489, and miR-455 influence cardiac fibrosis [39,40,41]. However, the roles of noncoding RNAs in arachnoid fibrosis and hydrocephalus remain unclear. Due to the limitation of this study, we have not paid attention to LncRNA research. The possible relationship between lncRNA and hydrocephalus still needs more work to verify in the future.

Increasing evidence has shown that miRNAs play an important role in fibrosis. Several previous studies have demonstrated that miR-30a can negatively regulate fibrosis in the myocardium [42], pulmonary interstitium[43], peritoneum [44], and so on [45,46,47], which is related to snail 1, TET1, CTGF, and TGF-β1. For example, overexpression of miR-133a can protect myocardium from fibrosis [20]. TGF-β1 promotes collagen expression and renal and orbital fibrosis by inhibiting miR-29 [21,48]. Liver fibrosis led to the downregulation of miR-150 and miR-194 in hepatic stellate cells, and overexpression of both reduced the activation of hepatic stellate cells [22]. Hsa-miR-4274, miR-9, miR-17, miR-26a, and other miRNAs may be related to hydrocephalus [49]. However, the relationship between miR-30a, arachnoid fibrosis, and even hydrocephalus had not been determined before. Here, we demonstrated that miR-30a can negatively regulate arachnoid fibrosis. The relationship between miR-30a and hydrocephalus, which is closely related to fibrosis, has not been studied before.

Several potential limitations of this experiment should be mentioned. First, the progression of PHH in rats is similar but not identical to PHH in humans. In addition, the relationship between TGF-β1 and miR-30a still needs further investigation. Moreover, in addition to the effect of miR-30a on the TRAF3IP2/AP-1 axis, the possible regulation of miR-30a on other factors still needs to be studied.

## 5 Conclusion

**Figure 8:**
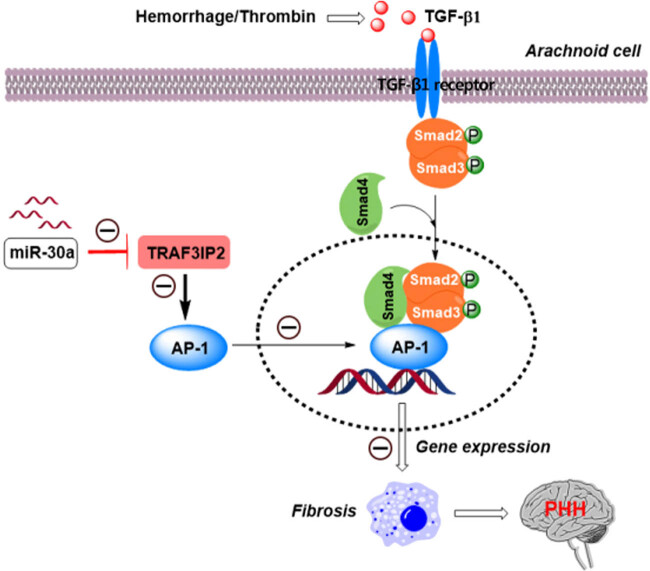
Scientific hypothesis. Decreased MiR-30a promotes TGF-β1-mediated arachnoid fibrosis in PHH.

In the pathogenesis of PHH, thrombin induces TGF-β1 activation and induces the expression of downstream fibrotic genes, which finally promoting arachnoid fibrosis and the hydrocephalus formation after hemorrhage. MiR-30a inhibits the transcriptional function of AP-1 by decreasing TRAF3IP2 expression, which finally can block arachnoid fibrosis and hydrocephalus formation after hemorrhage (Figure 8).
